# Expectant Management of a Critically Ill Pregnant Patient with COVID-19 with Good Maternal and Neonatal Outcomes

**DOI:** 10.1155/2020/8891787

**Published:** 2020-11-25

**Authors:** Farah Alsayyed, Victoria Hastings, Sanford Lederman

**Affiliations:** Division of Obstetrics and Gynecology, New York Presbyterian-Brooklyn Methodist Hospital, Brooklyn, NY, USA

## Abstract

**Background:**

Coronavirus Disease 2019 (COVID-19) is responsible for a global pandemic that has significantly affected New York City. There is limited data about COVID-19 infection in pregnancy, especially in critically ill patients.

**Case:**

A 30-year-old female who presented at 26 weeks gestation with acute severe respiratory distress that required intubation and intensive care unit (ICU) admission. We had a high suspicion of COVID-19 disease despite repeated negative SARS-CoV-2 PCR testing, with eventual positive COVID IgG antibody testing. Through an integration of obstetrical knowledge, critical care, and comparing outcomes from similar cases in the literature, we decided to expectantly manage her pregnancy and did not recommend administration of antenatal steroids. She was extubated after 23 days of mechanical ventilation and recovered from her respiratory illness. She had a full-term spontaneous vaginal delivery of a baby boy at 39 weeks gestation with excellent maternal and fetal outcomes at delivery.

**Conclusion:**

In the face of COVID-19, a new disease with unclear maternal and fetal outcomes to date, a collaboration of care teams is essential to navigate through the challenging decisions made, including timing of delivery, treatment options, and administration of steroids. Our paper is unique as there is no other published case report of a critically ill pregnant patient with COVID-19 in which delivery was deferred, and a full recovery was observed, with a vaginal delivery at term.

## 1. Introduction

Coronavirus Disease 2019 (COVID-19) is a respiratory illness caused by the SARS-CoV-2 virus responsible for a global pandemic that emerged in Wuhan China in December 2019. In the United States, there are currently a total of 39, 857 cases of pregnant women with COVID-19, with 8284 hospitalized patients and an approximate rate of 21% ICU admissions [1]. New York City was one of the first states overwhelmed by the pandemic. Across the New York Presbyterian Hospital system, universal screening was implemented for all admitted pregnant patients. Between March 22nd 2020 and May 1st 2020, 271 out of 2256 pregnant women admitted tested positive for SARS-CoV-2 (12.1%), of which 70 (3.1%) were symptomatic and 17 (0.7%) were admitted to the intensive care unit (ICU). None of these patients were intubated; however, these statistics do not include patients with suspected COVID-19 despite negative SARS-CoV-2 PCR testing.

The anatomical and physiological adaptations that occur in pregnancy necessitate careful multidisciplinary management strategies for COVID-19 patients. Several studies have been published which outline approaches to management of these patients, and the majority were delivered via cesarean section [2–5]. We present a case of a critically ill pregnant patient suspected to have COVID-19 who was admitted to a tertiary care hospital in Brooklyn. The patient provided written consent for publication. Antenatal management decisions in this case differ from those published previously.

## 2. Case Presentation

**Figure 1:**
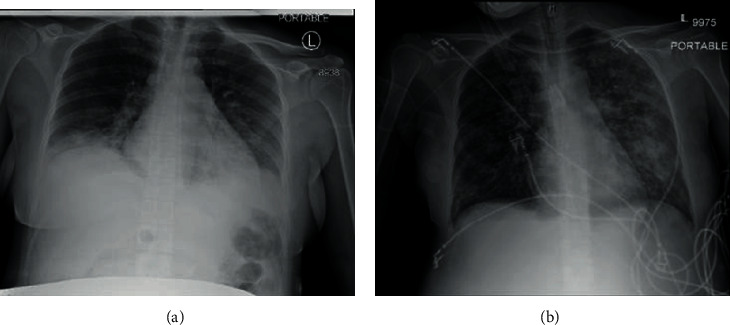
(a) Chest X-ray on admission, showing multifocal consolidative airspace opacities in lung bases bilaterally concerning multifocal pneumonia, possibly secondary to a viral pathogen. (b) Chest X-ray on HD 16 demonstrating generalized patchy bilateral lung airspace disease, with dense opacification and prominent interstitial lung markings representing the sequelae of ARDS.

A 30-year-old Chinese female, G3P2002 at 26 weeks and 2 days gestation, presented to the Emergency Department complaining of shortness of breath and a dry cough that started one week prior. She had no sick contacts or recent travel history and denied obstetrical complaints. Her history was significant for chronic hepatitis B and two prior vaginal deliveries. She appeared critically ill, with vitals as follows: febrile to 39.3°C, tachycardic to 128 beats per minute, tachypneic with a respiratory rate of 22 breaths per minute, hypotensive to 88/55 mmHg, and her oxygen saturation was 93% on room air. Pertinent laboratory findings included WBC 7.0 K/*μ*L, an initial neutrophil–lymphocyte ratio of 7.25, hemoglobin/hematocrit of 9.1 g/dL/28.1%, ESR of 50 mm/hr, procalcitonin of 178 pg/mL, CRP level of 69.6 mg/L, AST 14, ALT 32, and a BUN/creatinine ratio of 4 mg/dL/0.59. Her chest X-ray showed evidence of multifocal pneumonia (Figure 1). On initial obstetrical evaluation, a sonogram confirmed a male fetus in cephalic presentation, with an estimated fetal weight of 910 g. The biophysical profile (BPP) score was 8/8. She was admitted for management of sepsis secondary to suspected COVID-19 infection.

This patient underwent a septic work up including NP SARS-CoV-2 RT-PCR testing, respiratory viral panel, MRSA culture, legionella and streptococcus pneumoniae testing, blood cultures, and a urine culture. There was no clear etiology for her hypoxia, including negative SARS-CoV-2 testing, but was significant for asymptomatic bacteriuria, which was treated with cefpodoxime. Given the high clinical suspicion of COVID 19, SARS-CoV-2 testing was repeated but remained negative.

Within the first two days of admission the patient continued to deteriorate with increased work of breathing, increased oxygen requirements, and mixed respiratory and metabolic acidosis. On hospital day (HD) 3 her WBC increased to 10.6 K/*μ*L and trended to 21.6 K/*μ*L by HD9. The neutrophil to lymphocyte ratio was 16.41, lymphocyte percentage was 5%, and CRP was 187 mg/dL. She received ceftriaxone and was started on azithromycin for presumed community acquired pneumonia. She was evaluated by the critical care team for acute hypoxemic respiratory failure despite treatment with a nonrebreather mask and high-flow oxygen and was intubated on hospital day (HD) 4 with transfer to the ICU. A third SARS-CoV-2 test remained negative.

An interdisciplinary team including medicine, pulmonary, anesthesia, neonatology, and obstetrics with maternal fetal medicine guided clinical decision-making. It was felt that delivery would not provide maternal or fetal benefit; therefore, magnesium sulfate and antenatal steroids were not given.

In the ICU, she received a norepinephrine drip due to hemodynamic instability, propofol for sedation purposes, rocuronium for paralysis, and insulin drip for glycemic control, and heparin prophylaxis was initiated. She received 50 mg of methylprednisolone four times daily for 25 days, which was tapered over several days. Her acidosis was addressed and corrected with a goal pH of 7.4-7.47. She exhibited persistent leukocytosis, and her antibiotic coverage was changed to meropenem. She intermittently required blood transfusions for anemia to maintain a hemoglobin level above 7 mg/dL. Initially, she failed to maintain good oxygenation with worsening radiographic findings, and her ventilation settings were adjusted accordingly with increasing PEEP settings (maximum set at 16). Two additional SARS-CoV-2 PCR tests were repeated and were still negative. The patient then underwent a bronchoscopy on HD 14 with a bronchoalveolar lavage, and the fluid culture yielded no growth.

The question regarding delivery versus expectant management was revisited multiple times, and maternal fetal medicine recommended that a cesarean section only be performed if the patient continued to deteriorate despite maximum ventilation settings. In terms of antenatal monitoring, we could not implement continuous electronic fetal monitoring due to lack of remote monitoring capabilities and training limitations of the ICU house staff. Instead, we performed daily biophysical profiles to assess fetal status. We observed a score of 4/8 on HD16 (points removed for breathing and movement) that was attributed to an increase in the propofol and rocuronium titrations. The ICU team subsequently decreased the dosing which resulted in improved findings on the biophysical profile 2 days later. A sonographic estimated fetal weight (EFW) was measured on HD20 at 1165 g (9.5% percentile by WHO fetal growth calculator). Umbilical artery Dopplers were performed twice weekly thereafter and remained normal. Her ventilation settings were slowly weaned down and she showed radiologic improvement. She was extubated on hospital day twenty-five.

She remained in the ICU for one week after her extubation and required high-flow oxygen that was slowly weaned down to a nasal cannula. She was transferred to the antepartum unit requiring 2 L/min oxygen on a nasal cannula. Sonographic EFW on HD33 was 1549 g (21 percentile by WHO fetal growth calculator). Meeting her recovery milestones, she was discharged home at 31 weeks gestation on HD 35 with no supplemental oxygen requirements. She was seen in the clinic 2 weeks after discharge and was doing very well. She then underwent testing for COVID-19 IgG antibodies and had a positive test result.

She was readmitted to the antepartum unit with preterm contractions and 3 cm cervical dilation at 34 weeks. She was given a course of betamethasone and monitored for preterm labor; she was discharged two days later after making no cervical change. She subsequently underwent weekly NST/BPPs (consistently 10/10) and monthly growths with an estimated fetal growth of 2962 g at 38 weeks and 4 days (28% by WHO fetal growth calculator). The patient was admitted to Labor and Delivery at 39 weeks and 0 days gestation in labor and had an uncomplicated normal spontaneous vaginal delivery of a live male neonate in cephalic presentation. Apgar scores were 9 and 9 at 1, and 5 minutes, respectively, the neonatal weight was 2830 g, with a base excess of -7.9 and an arterial pH of 7.18. The placental pathology findings included meconium staining, and patchy avascular villi suggestive of fetal vascular malperfusion. The mother and child were discharged home from the hospital two days after delivery with no complications.

## 3. Discussion

### 3.1. COVID-19 Testing

Despite a high clinical suspicion of COVID-19, our patient had five negative NP SARS-CoV-2 PCR tests during her admission. The optimal timing to perform serological testing to detect antibodies was an ongoing discussion with the infectious disease specialists. Furthermore, serological testing was limited initially, and protocols were directed towards patients with positive SAR-CoV2 PCR testing. She was tested 21 days after being discharged with positive COVID-19 IgG antibodies. The lack of a definitive diagnosis was a significant barrier to implementing management strategies, specifically with regards to her triage. She was initially cared for in a COVID ICU, then transferred to a non-COVID ICU; however, strict precautions were adhered to by all staff due to ongoing suspicion.

The RT-PCR is widely adopted as the standard diagnostic method of SARS-CoV-2; however, the novelty of COVID-19 and its rapid spread limit the data available to determine its sensitivity. False negative rates of the RT-PCR test ranges from 17 to 63%. [6] One case report describes three negative nasopharyngeal RT-PCR tests with a positive fourth RT-PCR test of expectorated sputum [7]. Serological testing may be helpful in diagnosing COVID-19 in cases with negative RT-PCR testing. Long et al. report four out of 52 patients suspected to have COVID-19 had virus-specific IgG or IgM despite sequential negative viral RNA testing. They also report 100% seroconversion within 19 days, with measurable antibodies as early as 2-4 days [8]. As prompt diagnosis is crucial to appropriate management, serological testing should be considered early on and may need to be repeated if negative prior to 19 days after symptom onset [7].

Interestingly, chest CTs have also been studied as an adjuvant to the RT-PCR testing and may even be considered a primary tool for COVID-19 detection [9]. One study correlating CT chest images with RT-PCR testing found that in patients with negative RT-PCR results, 75% (308/413) had positive chest CT findings with 48% highly likely cases and 33% probable cases. In the setting of pregnancy, CT scans are only performed when the benefit outweighs the risk. Given the high clinical suspicion of COVID-19, a CT scan would not have altered management and was not performed.

### 3.2. Neutrophil–Lymphocyte Ratio

The neutrophil to lymphocyte ratio (NLR) is a marker of systemic inflammation used in various diseases and has been studied both as a diagnostic and prognostic marker in COVID-19. NLR has been shown to be significantly higher in moderate, severe, critical, and death groups than those of the healthy groups, and the lymphocyte percentages were significantly lower than healthy controls (the definitions of the patient's clinical status based on the trial version 7 protocol [10]). The combination of NLR and an elevated CRP for detecting COVID-19 had a sensitivity and specificity of 77.5% and 98%, respectively, in a study of 191 patients [11]. A study investigated the dynamic changes of the NLR in patients with COVID-19 who survived and those who did not to determine its prognostic value. The initial and peak NLR were collected and were significantly lower than those in deceased patients (*p* < 0.001). The initial median NRL in the deceased group was 14.96, and the peak was 46.58, with critical values of initial NRL 7.13 and peak NRL of 14.31. [12] Our patient who met severe and critical criteria had an initial NRL of 7.25, CRP of 69.9 mg/L, lymphocyte percentage of 5% and a peak NRL 16.41. The NLR and CRP were both highly suggestive of a diagnosis COVID-19. The NLR was also above the quoted critical values; however, her initial and peak NRL fell below the medians of the deceased groups, which is reflected by her survival. The emergence of NLR studies has added a valuable tool for clinicians that may face a similar dilemma of negative SAR-CoV2 PCR testing. Its prognostic value in setting of pregnancy may help navigate the conversation of fetal delivery that is often dependent on maternal status.

### 3.3. Delivery versus Expectant Management

Throughout this patient's ICU course, multiple multidisciplinary meetings were held to collaborate and decide on the mode of delivery as there is limited guidance in the literature. The largest published cohort of patients in the United States included 64 pregnant women who were critically ill with COVID-19 with 20 intubated patients in the second and third trimester, the majority of which were delivered via cesarean section (75%) in the setting of maternal respiratory distress [13]. Similarly, in a large cohort of 247 patients in the UK, 31 out of 40 (77%) of critically ill pregnant patients were also delivered [8]. Other case reports and case series with fewer reportable cases also showed that most interdisciplinary discussions ultimately led to the decision usually for a theoretical maternal benefit. [3, 14–18]

Per the American College of Obstetrics and Gynecology (ACOG) guidelines on critical care in pregnancy, there is limited evidence regarding the physiological benefit of performing a cesarean section to relieve maternal aortocaval pressure for patients on a ventilator in the setting of respiratory illness, especially in instances of severe prematurity [19]. In pregnancy, an increase in the minute ventilation and the resultant hyperventilation causes a decrease in the functional residual capacity (FRC) by approximately 20-30%. In addition, the diaphragm rises by 4 cm in the third trimester, which further reduces FRC. [15, 16, 20] This may lead to rapid deterioration in oxygenation in the setting of respiratory disease, which we witnessed within the first few days of our patients admission. After delivery, the minute ventilation returns to baseline within several weeks and decompression normalizes lung volumes. It is not clear that these changes translate to survival benefit in critically ill pregnant patients [2].

Conversely, it is well documented that a cesarean section is a major stressor. In the more immediate setting, it can result in fluid shifts with possible pathologic retention of fluid in septic pregnant women undergoing an unscheduled cesarean delivery [21, 22]. In the postpartum setting, cesarean sections increase the risk of infection and thromboembolic events, especially in the critically ill [21, 23]. Our concern was potential clinical deterioration postoperatively. Alternatively, the clinical worsening of patients as seen in one case series [3] may be due the pathophysiological course of COVID-19 which is not fully understood yet. The decision of whether to deliver these patients needs to weigh both maternal and fetal risks and benefits.

In our case, concerns of severe prematurity at 26 weeks and the potentially challenging maternal postoperative recovery were the factors that led to the decision to continue her pregnancy at the time of her intubation. Interdisciplinary meetings were held daily to reassess her constantly evolving clinical status. During her ICU course, her respiratory status initially worsened, but eventually stabilized before improving. While there was still capacity to increase her mechanical ventilatory support, and she continued to be stable, we continued to expectantly manage her pregnancy and perform daily biophysical profiles. We are able to report excellent maternal outcomes and neonatal outcomes after a vaginal delivery at full term with no postpartum complications. Interestingly, the placenta showed evidence of fetal vascular malperfusion, seen in 45% of 20 placental pathologies of COVID-19 patients [24]. The significance of this is still unclear.

### 3.4. Steroids

There is conflicting data regarding the risks and benefits of high-dose corticosteroid administration in critically ill COVID-19 patients. There is limited data, which does not include pregnant women, that shows worse outcomes with corticosteroid use [5]. However, corticosteroids used for obstetrical purposes differ from corticosteroid use in COVID-19 studies in two important ways. First, the dosages of betamethasone and dexamethasone are comparably smaller than the dose of methylprednisolone (one-fourth to one-tenth the amount). Secondly, steroids for fetal lung maturity are administered over 24 hours whereas the course of methylprednisolone can be administered over weeks [13]. For these reasons, antenatal corticosteroids are thought to be relatively safe, and the risk of neonatal distress syndrome can be significant in the early preterm period. ACOG recommends administration of antenatal corticosteroids for pregnant women less than 34 weeks gestational age if delivery is imminently indicated. As we did not anticipate delivery in this case, betamethasone was not administered at the time of the patients' intubation.

### 3.5. Treatment/Medication Safety in Pregnancy

The accurate diagnosis of COVID-19 in pregnant patients has the added implication of drug safety in pregnancy. Drugs of interest in the treatment of COVID-19 include hydroxychloroquine and antiviral medications such as remdesivir, lopinavir-ritonavir, and oseltamivir. There are emerging clinical trials that study the efficacy of these drugs in the treatment of COVID-19, but few studies that include pregnant women.

Of these treatments, remdesivir, a nucleotide analogue used in the treatment of Ebola, is the only medication that has shown activity against SARS-CoV-2 in patients with severe disease. However, fetal toxicity and placental transmission of the drug have not been studied [25]. Hydroxychloroquine had been widely implemented in hospital protocols. Based on studies of rheumatological diseases in pregnancy with comparable doses, hydroxychloroquine is known to cross the placenta with a low risk of teratogenicity. [2, 20, 26] However, it was eventually revoked by the FDA when it showed no benefit in treating COVID-19 and potential harm [27].

Studies regarding the efficacy of these medications in treating COVID-19 are so far inconsistent; the evidence mainly stems from retrospective cohorts, and randomized control trials are still in preliminary phases. Nonetheless, they have been incorporated into some hospital protocols due to potential benefits and due to the exhaustion of standard interventions in rapidly deteriorating patients. At the time that our patient was admitted, the current body of evidence had not strongly supported the use of hydroxychloroquine in COVID-19 patients, and it was not administered to our patient, and remdesivir was not yet FDA approved for COVID-19. Our patient did receive high-dose steroids for worsening cardiovascular status. She showed improvement and hemodynamic stability, and completed a 25-day course.

## 4. Conclusion

An interdisciplinary and individualized approach should be implemented with regard to the management of severe COVID-19 in pregnancy. With limited data and evolving guidelines, we carefully weighed the benefits of delivery against the risks of a cesarean section and decided not to deliver with a favorable outcome and reassuring fetal status. Future studies are needed to investigate the effect of delivery on ventilation settings, specifically the efficacy of treatments and fetal outcomes.

## Data Availability

The data included in this study includes laboratory results and images retrieved from the patient's medical chart.

## Additional Points

*Teaching Points*. The majority of pregnant patients who are critically ill with COVID-19 undergo cesarean section for presumed maternal benefit; here, we illustrate a case of expectant management resulting in successful extubation and eventual vaginal delivery at term to a healthy infant. Treatment recommendations for critically ill pregnant women with COVID-19 remain unclear, extrapolating largely from the nonpregnant population Antenatal steroids for fetal lung maturity should be administered to critically ill pregnant patients in the event delivery is imminent.
